# Development and validation of a metabolite index for obstructive sleep apnea across race/ethnicities

**DOI:** 10.1038/s41598-022-26321-9

**Published:** 2022-12-16

**Authors:** Ying Zhang, Debby Ngo, Bing Yu, Neomi A. Shah, Han Chen, Alberto R. Ramos, Phyllis C. Zee, Russell Tracy, Peter Durda, Robert Kaplan, Martha L. Daviglus, Stephen S. Rich, Jerome I. Rotter, Jianwen Cai, Clary Clish, Robert Gerszten, Bruce S. Kristal, Sina A. Gharib, Susan Redline, Tamar Sofer

**Affiliations:** 1 Division of Sleep Medicine and Circadian Disorders, Department of Medicine, Brigham and Women’s Hospital, Boston, MA 02115 USA; 2 Division of Pulmonary, Critical Care and Sleep Medicine, Beth Israel Deaconess Medical Center, Cardiovascular Institute, Boston, MA 02215 USA; 3 Department of Epidemiology, Human Genetics and Environmental Sciences, School of Public Health, Human Genetics Center, The University of Texas Health Science Center at Houston, Houston, TX 77030 USA; 4 Department of Medicine, Albert Einstein College of Medicine, New York, NY 10029 USA; 5 Center for Precision Health, School of Biomedical Informatics, The University of Texas Health Science Center at Houston, Houston, TX 77030 USA; 6 Sleep Medicine Program, Department of Neurology, University of Miami Miller School of Medicine, Miami, FL 33136 USA; 7 Division of Sleep Medicine, Department of Neurology, Northwestern University, Chicago, IL 60611 USA; 8 Department of Pathology Laboratory Medicine, Larner College of Medicine, University of Vermont, Burlington, VT 05405 USA; 9 Department of Epidemiology & Population Health, Albert Einstein College of Medicine, Bronx, NY 10461 USA; 10 Department of Preventive Medicine, Northwestern University Feinberg School of Medicine, Chicago, IL 60612 USA; 11 Center for Public Health Genomics, University of Virginia, Charlottesville, VA 22908 USA; 12 Department of Pediatrics, The Institute for Translational Genomics and Population Sciences, The Lundquist Institute for Biomedical Innovation at Harbor-UCLA Medical Center, Torrance, CA 90502 USA; 13 Department of Biostatistics, Collaborative Studies Coordinating Center, University of North Carolina at Chapel Hill, Chapel Hill, NC 27599 USA; 14 Metabolite Profiling Platform, Broad Institute of MIT and Harvard, Cambridge, MA 02142 USA; 15 Cardiovascular Research Center, Massachusetts General Hospital, Harvard Medical School, Boston, MA 02115 USA; 16 Department of Medicine, Sleep and Circadian Disorders, Harvard Medical School, Boston, MA 02115 USA; 17 Department of Medicine, Division of Sleep Medicine and Circadian Disorders, Brigham and Women’s Hospital and Harvard Medical School, Boston, MA 02115 USA; 18 Division of Pulmonary, Critical Care, and Sleep Medicine, Department of Medicine, University of Washington, Seattle, WA 98195 USA; 19 Department of Biostatistics, Harvard T.H. Chan School of Public Health, Boston, MA 02115 USA

**Keywords:** Cardiovascular diseases, Biomarkers, Epidemiology

## Abstract

Obstructive sleep apnea (OSA) is a common disorder characterized by recurrent episodes of upper airway obstruction during sleep resulting in oxygen desaturation and sleep fragmentation, and associated with increased risk of adverse health outcomes. Metabolites are being increasingly used for biomarker discovery and evaluation of disease processes and progression. Studying metabolomic associations with OSA in a diverse community-based cohort may provide insights into the pathophysiology of OSA. We aimed to develop and replicate a metabolite index for OSA and identify individual metabolites associated with OSA. We studied 219 metabolites and their associations with the apnea hypopnea index (AHI) and with moderate-severe OSA (AHI ≥ 15) in the Hispanic Community Health Study/Study of Latinos (HCHS/SOL) (n = 3507) using two methods: (1) association analysis of individual metabolites, and (2) least absolute shrinkage and selection operator (LASSO) regression to identify a subset of metabolites jointly associated with OSA, which was used to develop a metabolite index for OSA. Results were validated in the Multi-Ethnic Study of Atherosclerosis (MESA) (n = 475). When assessing the associations with individual metabolites, we identified seven metabolites significantly positively associated with OSA in HCHS/SOL (FDR *p* < 0.05), of which four associations—glutamate, oleoyl-linoleoyl-glycerol (18:1/18:2), linoleoyl-linoleoyl-glycerol (18:2/18:2) and phenylalanine, were replicated in MESA (one sided-*p* < 0.05). The OSA metabolite index, composed of 14 metabolites, was associated with a 50% increased risk for moderate-severe OSA (OR = 1.50 [95% CI 1.21–1.85] per 1 SD of OSA metabolite index, *p* < 0.001) in HCHS/SOL and 55% increased risk (OR = 1.55 [95% CI 1.10–2.20] per 1 SD of OSA metabolite index, *p* = 0.013) in MESA, both adjusted for demographics, lifestyle, and comorbidities. Similar albeit less significant associations were observed for AHI. Replication of the metabolite index in an independent multi-ethnic dataset demonstrates the robustness of metabolomic-based OSA index to population heterogeneity. Replicated metabolite associations may provide insights into OSA-related molecular and metabolic mechanisms.

## Introduction

Obstructive sleep apnea (OSA) is a common disorder characterized by recurrent episodes of upper airway obstruction during sleep resulting in oxygen desaturation and sleep fragmentation^1^. While highly prevalent in the population^2^, OSA is severely under-diagnosed^3^, especially in women^4^. For example, only 1.3% of participants in the Hispanic Community Health Study/Study of Latinos (HCHS/SOL) and 7–15% of participants in the Multi-Ethnic Study of Atherosclerosis (MESA) reported a previous OSA diagnosis; in MESA, underdiagnosis was highest among race/ethnic minorities^5,6^. The pathophysiology underlying OSA is multifaceted, which includes obesity, craniofacial structure, upper airway neuronal control, ventilatory control, and inflammation, among others^7^. OSA is also associated with increased risk of adverse health outcomes, including hypertension, cardiovascular disease, diabetes, and early mortality^8–11^.

Metabolomics is the study of small biochemical compounds at a large scale^12^. As a growing number of large metabolomics datasets become available, metabolites are being increasingly leveraged for biomarker discovery and evaluation of disease processes and progression^13,14^. In particular, studying metabolite associations with OSA may improve our understanding of the pathophysiology of OSA. However, research in this area has been limited by the relatively small number of participants that have undergone both overnight sleep studies and metabolomic profiling, the lack of representativeness of the study subjects selected solely in clinical encounters, and the limited metabolite panels used by many targeted metabolomics platforms^15,16^.

**Figure 1:**
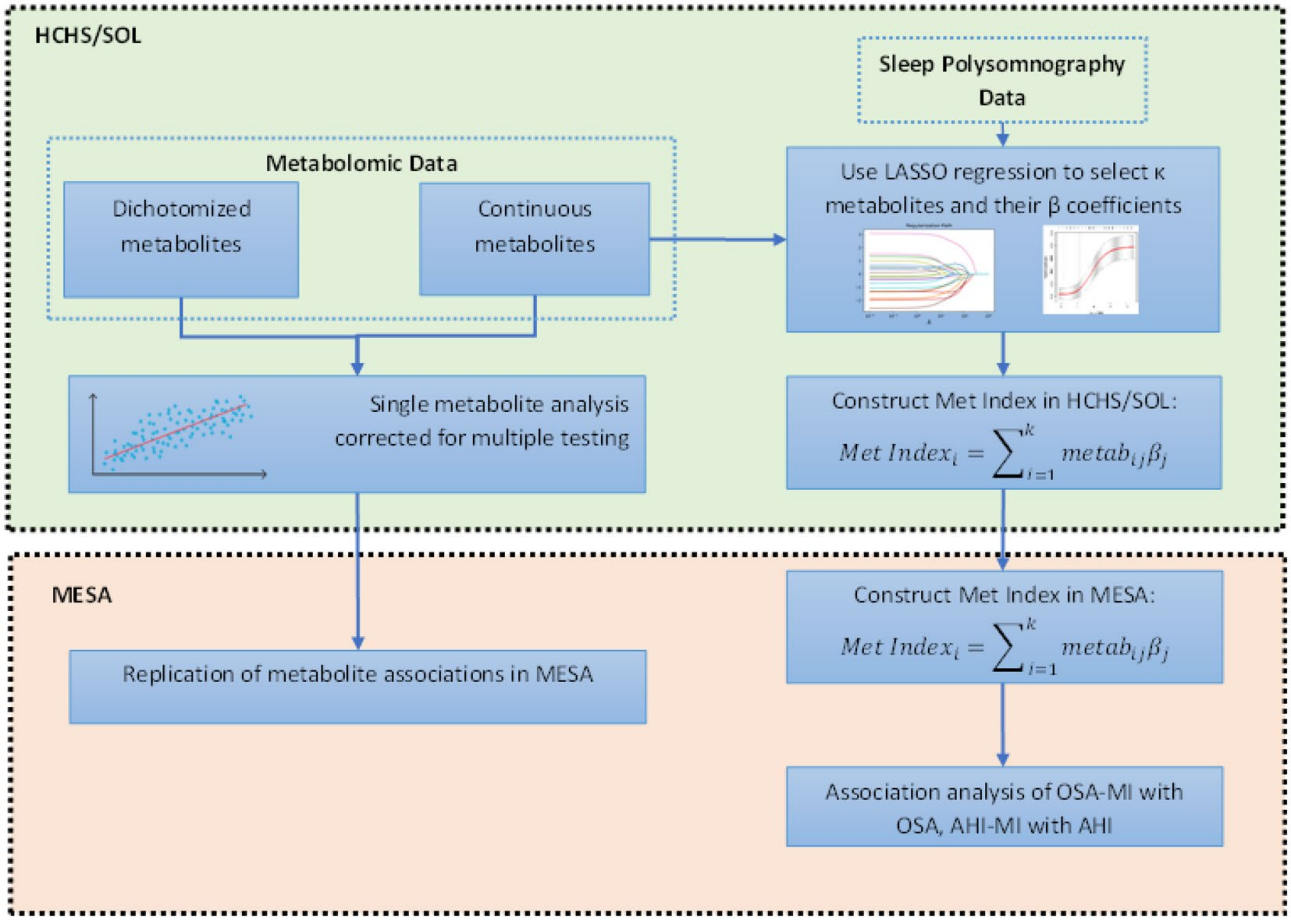
Study design flow chart. Abbreviations: *LASSO* least absolute shrinkage and selection operator, *HCHS/SOL* the Hispanic Community Health Study/Study of Latinos, *MESA* the Multi-Ethnic Study of Atherosclerosis, *Met Index* metabolite index, *metab* metabolite.

Here, we study metabolite associations with OSA in the HCHS/SOL, one of the largest multi-center cohorts with diverse participants from a rapidly growing minority group in the US: Hispanics/Latinos. We then test these associations for replication in MESA, a multi-ethnic community-based cohort. Our study follows the design described in Fig. 1. To start, we study associations of individual metabolites with a measure of moderate to severe OSA, defined by the apnea hypopnea index (AHI) ≥ 15, as well as by associations with continuously measured AHI. Next, we develop a metabolite index for OSA by aggregating together multiple metabolites as a potential biomarker for OSA. We then validate its association with OSA in MESA. This process is repeated for the AHI. Because OSA has different characteristics across genders^17^, in secondary analyses we additionally studied gender-specific metabolite indices.

## Methods

### The Hispanic Community Health Study/Study of Latinos

The HCHS/SOL is a community-based cohort study of 16,415 self-identified Hispanic/Latino persons from diverse Hispanic/Latino backgrounds (Mexicans, Puerto Ricans, Cubans, Central Americans, Dominicans, and South Americans)^18^. Participants 18–74 years of age at their baseline examination were recruited through a stratified multistage area probability sample design from four communities: San Diego, California; Chicago, Illinois; The Bronx, New York; and Miami, Florida. The baseline examinations occurred in June 2008–July 2011 and included assessment of OSA using a validated Type 3 home sleep apnea test (ARES Unicorder 5.2; B-Alert, Carlsbad, CA) that measured nasal air-flow, position, snoring, heart rate and oxyhemoglobin saturation^19^ as previously described^5^ in 14,440 individuals. The current cross-sectional analysis included 3507 of these participants who also had blood assessed for metabolomic measures. Our primary analysis focused on a metabolomics-based biomarker for moderate or severe OSA defined as AHI ≥ 15, with events defined as apneas or hypopneas with at least 50% cannula flow reduction for a minimum duration of 10 s, with ≥ 3% oxygen desaturation. The sleep study was conducted within the week following the baseline exam in which blood was collected and used for metabolite quantification. The HCHS/SOL was approved by the institutional review boards (IRBs) at each field center, where all participants gave written informed consent, and by the Non-Biomedical IRB at the University of North Carolina at Chapel Hill, to the HCHS/SOL Data Coordinating Center. All IRBs approving the study are: Non-Biomedical IRB at the University of North Carolina at Chapel Hill. Chapel Hill, NC; Einstein IRB at the Albert Einstein College of Medicine of Yeshiva University. Bronx, NY; IRB at Office for the Protection of Research Subjects (OPRS), University of Illinois at Chicago. Chicago, IL; Human Subject Research Office, University of Miami. Miami, FL; Institutional Review Board of San Diego State University, San Diego, CA. All methods and analyses of HCHS/SOL participants’ materials and data were carried out in accordance with human subject research guidelines and regulations.

### HCHS/SOL metabolomic profiling

Fasting blood samples were collected at the baseline examination. Of all HCHS/SOL participants from the baseline examination who also had genetic data, 3968 individuals were selected at random for metabolomics assessment. The samples were processed, and serum was stored at − 70 °C since collection. The metabolomic profiling was conducted at Metabolon (Durham, NC) with Discovery HD4 platform in 2017. Serum metabolites were quantified with untargeted, liquid chromatography-mass spectrometry (LC–MS)-based quantification protocol^20^. The platform captured a total of 1136 metabolites, including 782 known and 354 unknown (unidentified) metabolites.

### The Multi-Ethnic Study of Atherosclerosis

MESA is a cohort study designed to study risk factors for clinical and subclinical cardiovascular diseases in four racial/ethnic groups^21^. The study began in July 2000 and recruited 6,814 adults free of clinical CVD and aged 45–84 years from 6 centers: Baltimore, MD; Chicago, IL; Los Angeles, CA; New York, NY; Saint Paul, MN; and Winston-Salem, NC. Participants were continued to be studied through subsequent follow-up exams. Of the 4077 participants who attended Exam 5 (2010–2012), 2261 participated in the MESA Sleep ancillary study (2010–2013) which occurred at a mean interval of 301 days (range 0–1, 024 days) after the MESA Exam 5. The sleep study was only conducted on participants who were not receiving sleep apnea treatment. As reported before^6^, participants in the Sleep Exam were generally similar to non-participants. OSA was assessed with Type II in-home polysomnography. AHI was defined as the total number of apnea and hypopneas with at least 30% reduction in the nasal flow signal and with ≥ 3% oxygen desaturation per hour of sleep. Metabolomic data were collected on 1,000 randomly selected participants from the fasting samples collected during the MESA Exam 5 core exam. Of these, 475 participants also had sleep measures and are included in this analysis. All MESA participants provided written informed consent, and the study was approved by the Institutional Review Boards at The Lundquist Institute (formerly Los Angeles BioMedical Research Institute) at Harbor-UCLA Medical Center, University of Washington, Wake Forest School of Medicine, Northwestern University, University of Minnesota, Columbia University, and Johns Hopkins University. All methods and analyses of MESA participants’ materials and data were carried out in accordance with human subject research guidelines and regulations.

### MESA metabolomic profiling

Metabolite profiling was performed using liquid chromatography tandem mass spectrometry (LC–MS). Positive ion mode profiling of water-soluble metabolites and lipids was performed using LC–MS systems comprised of Nexera X2 U-HPLC (Shimadzu Corp.; Marlborough, MA) units coupled to a Q Exactive mass spectrometer (Thermo Fisher Scientific; Waltham, MA). Polar metabolites were analyzed using hydrophilic interaction liquid chromatography (HILIC) and lipids were analyzed separately using reversed phase C8 chromatography as described in detail previously^22^. Raw data were processed using TraceFinder 3.1 (Thermo Fisher Scientific; Waltham, MA) and Progenesis QI (Nonlinear Dynamics; Newcastle upon Tyne, UK). To measure organic acids and other intermediary metabolites in negative ionization mode, chromatography was performed using an Agilent 1290 infinity LC system equipped with a Waters XBridge Amide column, coupled to an Agilent 6490 triple quadrupole mass spectrometer. Metabolite transitions were assayed using a dynamic multiple reaction monitoring system. LC–MS data were analyzed with Agilent Masshunter QQQ Quantitative analysis software. Isotope labeled internal standards were monitored in each sample to ensure proper MS sensitivity for quality control. Pooled plasma samples were interspersed at intervals of 10 participant samples for standardization of drift over time and between batches. Additionally, separate pooled plasma was interspersed at every 20 injections to determine coefficient of variation for each metabolite over the run. Peaks were manually reviewed in a blinded fashion to assess quality. For each method, metabolite identities were confirmed using authentic reference standards or reference samples. Metabolites with poor peak quality and coefficients of variation greater than 30% averaged across batches were removed from analysis.

### Quality control of metabolites in HCHS/SOL and MESA

Missing metabolite values were addressed as described in Supplementary Fig. S1. In our discovery sample (HCHS/SOL), we excluded individuals with more than 25% missing metabolite values, and excluded metabolites with missing values for 75% or more individuals. For metabolites with more than 25% and less than 75% missing values, values were dichotomized as “observed” and “unobserved”. For metabolites with less than 25% missing values, we imputed the missing values using the minimum observed value of the metabolite in the sample, under the assumption that metabolites were not observed due to a technical detection limit.

Because our study design included validation analysis, we focused on metabolites available in both HCHS/SOL and MESA. Before any quality control (QC) methods were applied, 231 HCHS/SOL metabolites were mapped to 294 metabolites in MESA. The mapping of MESA to HCHS/SOL metabolites as well as to RefMet ID was done at the Clish Lab. MESA had multiple metabolites matched to a single HCHS/SOL metabolite in multiple instances because the same metabolite was measured via more than one platform used by MESA. In some cases, a single metabolite appears as two highly correlated ion features in the same MESA platform (e.g., some neutral lipids were measured as both sodium and ammonium adducts). Therefore, a single feature was mapped to the metabolite in HCHS/SOL while the redundant features were dropped according to the following principles: features with redundant ions were excluded; features with lower missingness and lower skewness were prioritized. After removing 60 such redundant features in MESA and applying QC methods based on the missingness in HCHS/SOL, 219 HCHS/SOL metabolites were mapped to 219 metabolites in MESA. Supplementary Table S1 provides the list of the 294 initially matched metabolites cross-referenced by RefMet ID and metabolite annotations including HMDB IDs provided by Metabolon, along with details regarding metabolite-specific QC resulting in the final list of one-to-one matched metabolites. The serum concentration values of the matched metabolites that were treated as continuous were rank-normalized.

Because MESA was a validation study, we only evaluated metabolites that were identified in the association analysis in HCHS/SOL. The missing data for these metabolites were always < 25%, so we treated these as continuous variables and imputed missing values with the minimum observed value in the MESA sample.

### Statistical analysis

Association analyses were based on three conceptual regression models: Model 1 (i.e. primary model) adjusted for demographic variables – age, gender, study center, Hispanic background (Mexicans, Puerto Ricans, Cubans, Central Americans, Dominicans, and South Americans and other/multi), and body mass index (BMI) in HCHS/SOL; age, gender, study site (two sites with low sample sizes were combined), race (White versus “Non-White”, which consists of Hispanic, Black and Chinese Americans), and BMI in MESA. Model 2 (i.e. lifestyle model) adjusted for demographic and lifestyle variables – alcohol use, cigarette use, total physical activity (MET-min/day), and diet (Alternative Healthy Eating Index 2010) in HCHS/SOL; alcohol use and cigarette use in MESA. Model 3 (i.e. lifestyle and comorbidity model) adjusted for demographic, lifestyle and comorbidity variables—indicators for diabetes, hypertension, fasting insulin, fasting glucose, HOMA-IR, HDL, LDL, total cholesterol, triglycerides, systolic blood pressure and diastolic blood pressure in HCHS/SOL; hypertension, fasting glucose, HDL, LDL, cholesterol, triglycerides, systolic blood pressure and diastolic blood pressure in MESA. We used complete data with respect to covariates, so that individuals with missing covariates were removed and models with more covariates usually had lower sample sizes.

### Single Metabolite Association (SMA) analysis for OSA and AHI

We tested the associations of each of 219 metabolites (both continuous and dichotomized metabolites) with moderate-severe OSA and AHI in HCHS/SOL. Each metabolite was the exposure in either linear or logistic regression (depending on the outcome) for each model. We accounted for the HCHS/SOL study design (sampling and clustering) and obtained representative effect estimates using survey regression implemented in the R *survey* package (4.0)^23^. We controlled the false discovery rate (FDR) using the Benjamini–Hochberg procedure^24^ and determined significant associations as those with FDR *p*-value < 0.05. We visualized the Spearman’s correlations among significant metabolites in HCHS/SOL. In the replication analysis, we tested the associations of these metabolites with OSA in logistic regression and with AHI in linear regression in MESA in model 1–3. We computed one-sided *p*-values guided by the estimated direction of the SMA analysis results in HCHS/SOL^25^, and determined replication if the one-sided *p*-value was < 0.05.

For the secondary analyses, gender-stratified analysis and interaction analysis between individual metabolite and gender were conducted to assess potential gender differences. We performed gender-stratified analysis regardless of interaction *p*-values because interaction models typically assume an additive difference in the metabolite association with the outcome between gender groups while all other model parameters are assumed identical. We also conducted the SMA analysis for OSA and AHI in the full set of 1136 metabolites from HCHS/SOL to encourage hypothesis generation by the research community.

### Metabolite indices construction and validation

We applied a LASSO logistic regression with moderate to severe OSA versus no or mild OSA (for brevity “OSA versus no OSA”), and linear regression with log-transformed AHI as log(AHI + 1), adjusted for the covariates in model 1 in HCHS/SOL. We included 209 continuous metabolites (not including the 10 dichotomized metabolites). We selected the LASSO tuning parameter by maximizing the area-under-curve for OSA, and minimizing the prediction error for AHI, in a tenfold cross-validation. LASSO metabolite indices were calculated as a weighted sum of the (normalized) metabolite serum concentrations, with weights being the metabolite coefficients from the LASSO regression. For interpretability of association, we then standardized or “z-scored” the indices by subtracting the sample mean and then dividing the resulting number by the sample standard deviation.

As secondary analyses, we constructed additional metabolite indices using the metabolites identified in the SMA analysis (FDR *p* < 0.05): (1) Using the effect size estimates from the SMA analysis as weights; (2) Fitting an un-penalized regression model using these metabolites and extracting the coefficients from the model as weights (SMA-GLM). The goal of the latter approach was to better account for correlations among metabolites. We then calculated the weighted sum of the normalized metabolites serum levels, which was then z-scored to generate two additional sets of metabolite indices.

To validate the metabolite index association with their outcomes, we constructed, when possible (i.e. when metabolites were identified in LASSO/SMA), the three types of metabolite indices in MESA by rank-normalizing the matched metabolites within MESA, and then summing them using the weights developed in HCHS/SOL. We then assessed the associations of the indices, z-scored using MESA-specific means and SDs, with their corresponding sleep traits (i.e. OSA, AHI) in model 1–3. In the secondary analyses we assessed (1) potential gender differences using gender-stratified LASSO, and, separately, gender-stratified association analysis for metabolite indices constructed based on combined genders; (2) potential temporal effect in MESA by adjusting for the time differences between the sleep study and the metabolite profiling; (3) potential racial/ethnic effects by limiting the study sample in MESA to Hispanics; (4) associations with other sleep disordered breathing phenotypes (i.e. the percentage of sleep time with oxyhemoglobin saturation below 90% (Pctlt90), minimum oxygen saturation, average oxygen saturation, and average respiratory event length). We also assessed the associations between metabolite indices quartiles and the corresponding sleep traits.

All analyses were done in R 3.6.3. The glmnet package (3.0)^26^ in R was used for the LASSO logistic regression. R code for constructing metabolite indices using metabolites and weights developed in this work are provided in Supplementary File 1.

## Results

### Participant characteristics

Table 1 characterizes the HCHS/SOL analytic sample and target population. The HCHS/SOL cohort included 3,507 participants, with a mean age of 41.72 years (SD = 15.4), of whom 50.7% were female and 10.2% were classified with moderate or severe OSA (AHI ≥ 15). Participants with OSA were more likely to be males, had a higher BMI, and were less likely to be never smokers compared to those without OSA. Individuals with OSA were also more likely to have comorbidities: 60% had hypertension and 34.3% had diabetes, compared to 27.8% with hypertension and 16.9% with diabetes in those without OSA. Supplementary Table S2 characterizes the 475 MESA participants with metabolomics, sleep, and required measured covariates from the validation dataset. Compared to HCHS/SOL, MESA participants were older (mean 68.45 years, SD = 9.33), with a higher proportion of females (56.2%). Reflecting their older age, more MESA participants had moderate or severe OSA (46.7%) compared to HCHS/SOL.

**Table 1:** Characteristics of Hispanics/Latinos represented by the HCHS/SOL study population.

Mean (SD)	Stratified by gender		Stratified by OSA status		Total
	Female	Male	AHI < 15	AHI ≥ 15	
n	2011	1496	3122	385	3507
Age (years)	42.22 (15.12)	41.20 (14.94)	40.29 (14.60)	54.20 (12.91)	41.72 (15.04)
Gender = Male (%)	0 (0.0)	1496 (100.0)	1252 (40.1)	244 (63.4)	1496 (49.3)
**Latino background (%)**					
Dominican	220 (10.9)	121 (8.2)	301 (9.6)	40 (10.4)	341 (10.7)
Central American	205 (10.2)	136 (5.7)	304 (9.7)	37 (9.6)	341 (6.4)
Cuban	279 (13.9)	279 (24.5)	479 (15.3)	79 (20.5)	558 (21.5)
Mexican	795 (39.5)	558 (35.7)	1221 (39.1)	132 (34.3)	1353 (36.7)
Puerto Rican	334 (16.6)	267 (17.8)	535 (17.1)	66 (17.1)	601 (16.4)
South American	121 (6.0)	86 (3.7)	187 (6.0)	20 (5.2)	207 (4.5)
Multi/other	57 (2.8)	49 (4.3)	95 (3.0)	11 (2.9)	106 (3.8)
**Alcohol drinking status (%)**					
Never	512 (25.5)	116 (8.1)	568 (18.2)	60 (15.6)	628 (16.3)
Former	698 (34.7)	447 (27.7)	1000 (32.0)	145 (37.7)	1145 (29.5)
current	800 (39.8)	933 (64.1)	1553 (49.8)	180 (46.8)	1733 (54.2)
**Smoking status (%)**					
Never	1382 (68.8)	710 (47.5)	1906 (61.1)	186 (48.3)	2092 (61.5)
Former	320 (15.9)	391 (26.2)	589 (18.9)	122 (31.7)	711 (16.2)
Current	308 (15.3)	394 (26.4)	625 (20.0)	77 (20.0)	702 (22.3)
BMI (kg/m^2^)	30.02 (6.90)	28.81 (5.34)	28.99 (6.09)	33.24 (5.97)	29.43 (6.21)
OSA status = OSA, AHI $\ge$ 15 (%)	141 (7.0)	244 (16.3)	0 (0.0)	385 (100.0)	385 (10.2)
AHI (3% desaturation)	3.64 (7.52)	8.01 (14.93)	2.60 (3.43)	33.80 (20.62)	5.79 (11.97)
Overnight Hypoxemia (> = 5% sleep < 90% saturation)	41 (2.0)	86 (5.7)	6 (0.2)	121 (31.4)	127 (3.2)
Diabetes status (ADA)^†^ = Yes (%)	376 (18.7)	283 (18.9)	527 (16.9)	132 (34.3)	659 (15.3)
Hypertension status^‡^ = Yes (%)	642 (31.9)	458 (30.6)	869 (27.8)	231 (60.0)	1100 (25.8)
Triglycerides (mg/dL)	115.91 (71.29)	144.00 (99.29)	124.97 (83.48)	171.88 (107.33)	129.77 (87.38)
HDL (mg/dL)	52.51 (12.73)	45.18 (11.90)	49.44 (13.03)	44.06 (10.11)	48.89 (12.86)
LDL (mg/dL)	119.63 (35.66)	122.36 (35.61)	120.43 (35.72)	125.75 (34.77)	120.97 (35.66)
Fasting glucose (mg/dL)	99.99 (32.62)	104.72 (34.52)	100.92 (31.83)	114.64 (44.85)	102.33 (33.65)
Fasting insulin (mU/L)	197.84 (147.49)	200.34 (142.42)	199.60 (145.42)	194.39 (141.28)	199.07 (145.00)
HOMA-IR	3.39 (3.72)	3.48 (5.41)	3.17 (3.26)	5.77 (10.53)	3.43 (4.63)
Total cholesterol (mg/dL)	195.40 (41.67)	196.06 (42.86)	194.82 (42.38)	203.65 (40.35)	195.72 (42.26)
Systolic blood pressure (mm Hg)	116.13 (17.69)	124.17 (15.57)	118.73 (16.51)	132.07 (18.06)	120.09 (17.15)
Diastolic blood pressure (mm Hg)	71.02 (10.53)	74.32 (10.92)	71.87 (10.60)	79.49 (10.61)	72.65 (10.85)

*Means and percentages were weighted using sampling weights to provides estimates of the HCHS/SOL target population characteristics.

^†^Baseline diabetes are based on American Diabetes Association definition (Diabetes Care 2010;33:S62-69), defined as fasting glucose ≥ 100 mg/dL, or post-OGTT glucose ≥ 140 mg/dL or A1C ≥ 5.7%, or use of anti-diabetic medication.

^‡^Baseline hypertension is defined as systolic or diastolic BP greater than or equal to 140/90 respectively, or current use of antihypertensive medications.

### Metabolite associations with OSA and AHI

**Figure 2:**
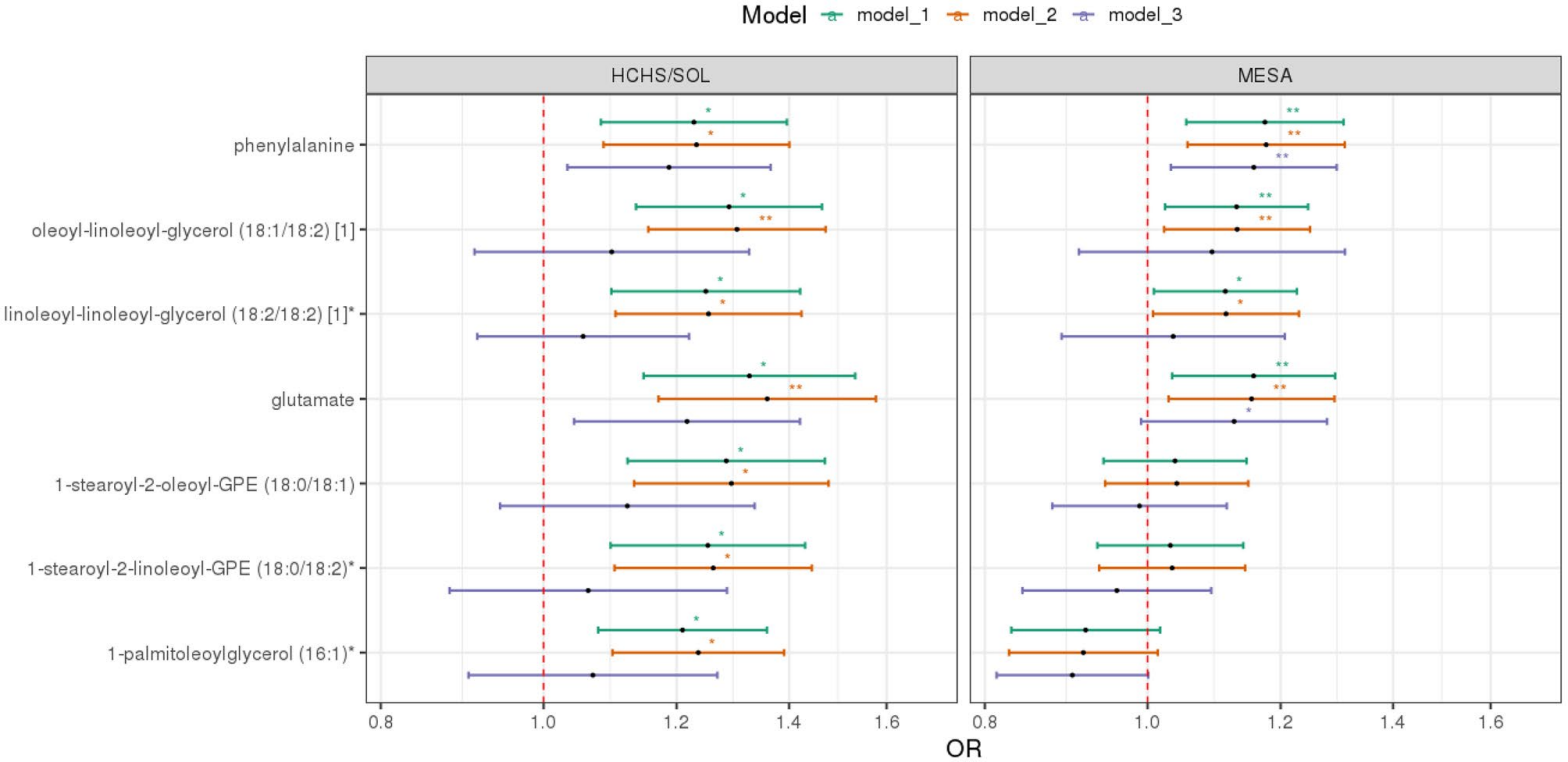
Estimated odds ratios of metabolites with significant FDR-adjusted *p*-value for OSA in HCHS/SOL and in MESA. *indicates FDR *p* < 0.05 in HCHS/SOL and one-sided *p* < 0.05 in MESA. **indicates FDR *p* < 0.01 in HCHS/SOL and one-sided *p* < 0.01 in MESA. In HCHS/SOL: Model 1 adjusted for age, gender, center, background, and BMI. Model 2 adjusted for age, gender, center, background, BMI, alcohol use, smoking status, physical activity and diet (AHEI 2010). Model 3 adjusted for age, gender, center, background, BMI, alcohol use, smoking status, physical activity, diet, diabetes, hypertension, fasting glucose, fasting insulin, HOMA IR, HDL, LDL, total cholesterol, triglycerides, systolic blood pressure and diastolic blood pressure. In MESA: Model 1 adjusted for age, gender, BMI, study site (site WFU and UCLA are combined due to low cell count), and race. Model 2 adjusted for age, gender, BMI, study site, race, alcohol use and smoking status. Model 3 adjusted for age, gender, BMI, study site, race, alcohol use, smoking status, hypertension indicator, fasting glucose, HDL, LDL, cholesterol, triglycerides, systolic blood pressure and diastolic blood pressure.

Table 2 shows the odds ratios corresponding to 7 metabolites associated (FDR *p* < 0.05) with OSA in HCHS/SOL adjusted for age, gender, BMI, study site/center, race/ethnicity). Figure 2 and Supplementary Table S3 show the lifestyle-adjusted model and comorbidity-adjusted model results for the 7 metabolites while results of all tested metabolites can be found in Supplementary Table S4. Among the 7 mapped metabolites in MESA, 4 metabolite associations had one-sided *p*-values < 0.05: glutamate, phenylalanine, linoleoyl-linoleoyl-glycerol (18:2/18:2), and oleoyl-linoleoyl-glycerol (18:1/18:2), all of which were associated with increased risk for OSA (Fig. 2). These associations also had FDR *p*-value < 0.05 in MESA. No metabolite was associated with AHI after multiple testing correction in HCHS/SOL (Supplementary Table S5). Also, no metabolite associations were detected at the FDR *p* < 0.05 level in minimally adjusted gender-stratified analyses in HCHS/SOL (Supplementary Tables S6–S9). Results for interaction analysis between metabolites and gender for OSA and AHI are provided in Supplementary Tables S10 and S11, respectively.

**Table 2:** Single metabolite associations with FDR-adjusted *p*-value < 0.05 in the discovery step.

Biochemical	Super-pathway	Sub-pathway	HMDB ID	HCHS/SOL			MESA				
				OR	Unadj-p^†^	FDR p	Platform	OR	Unadj-p^†^	1-sided p	1-sided FDR p
Glutamate	Amino Acid	Glutamate Metabolism	HMDB00148	1.33	1.36E–04	**0.014**	Amide-neg	1.16	1.11E–02	**0.006**	**0.019**
Phenylalanine	Amino Acid	Phenylalanine Metabolism	HMDB00159	1.23	1.63E–03	**0.049**	HIL-pos	1.17	3.55E–03	**0.002**	**0.014**
Linoleoyl-linoleoyl-glycerol (18:2/18:2) [1]*	Lipid	Diacylglycerol	HMDB07248	1.25	8.27E–04	**0.040**	HIL-pos	1.11	3.47E–02	**0.017**	**0.030**
Oleoyl-linoleoyl-glycerol (18:1/18:2) [1]	Lipid	Diacylglycerol	HMDB07219	1.29	8.90E-05	**0.014**	HIL-pos	1.13	1.51E–02	**0.008**	**0.019**
1-palmitoleoylglycerol (16:1)*	Lipid	Monoacylglycerol	HMDB11565	1.21	1.15E–03	**0.040**	C8-pos	0.92	9.96E–02	0.950	0.950
1-stearoyl-2-linoleoyl-GPE (18:0/18:2)*	Lipid	Phosphatidylethanolamine (PE)	HMDB08994	1.25	9.89E–04	**0.040**	HIL-pos	1.03	5.37E–01	0.269	0.314
1-stearoyl-2-oleoyl-GPE (18:0/18:1)	Lipid	Phosphatidylethanolamine (PE)	HMDB08993	1.28	2.77E–04	**0.019**	C8-pos	1.04	4.49E–01	0.225	0.314

*Metabolite with * indicates they were identified based on accurate mass data, retention time and mass spectrometry but not reference standards. Therefore, the verification is not as robust as metabolites without *.

^†^All results were adjusted for age, gender, BMI, study center, and Hispanic/Latino background in HCHS/SOL, and adjusted for age, gender, BMI, study site (site WFU and UCLA are combined due to low cell count), and race in MESA. Unadj-*p* is the abbreviations of raw *p* before the FDR correction. 1-sided p-values reflect the requirement of the same direction of associations in the discovery and validation datasets for concluding replication.

Significant values are in bold.

In the secondary analysis, the SMA analysis was performed in all metabolites assessed in HCHS/SOL– including 782 known and 354 unknown (unidentified) metabolites. Of these, 20 and 4 metabolites were found to be significantly associated with OSA and AHI (FDR *P* < 0.05), respectively (Supplementary Tables S12–S17).

### LASSO regression for joint selection and estimation of metabolite associations with OSA and AHI in HCHS/SOL

**Figure 3:**
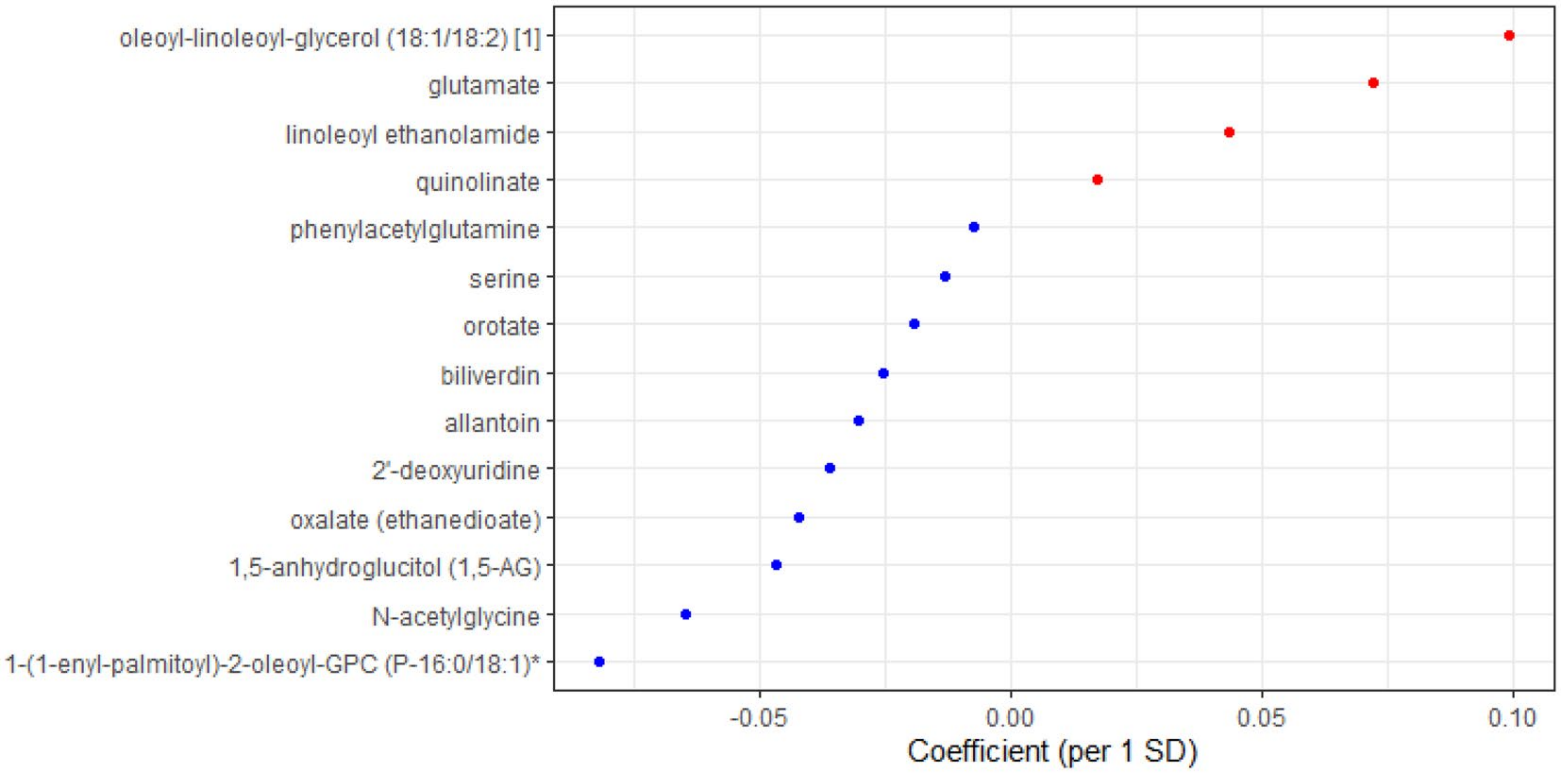
Coefficients for metabolites selected by LASSO (OSA model) in HCHS/SOL. Blue: coefficient < 0; Red: coefficient > 0. *OSA* moderate to severe obstructive sleep apnea (AHI ≥ 15), *LASSO* least absolute shrinkage and selection operator.

We used a LASSO regression to select a set of metabolites that jointly associated with sleep apnea traits in the HCHS/SOL. Among the 14 metabolites identified for OSA by LASSO (Fig. 3), there were one carbohydrate, one peptide, three amino acids, three lipids, three nucleotides, and three cofactors and vitamins. Biliverdin and serine were unique to the OSA metabolite index while the remaining 12 metabolites were shared between the OSA and AHI metabolite indices. A total of 41 metabolites were identified for AHI, among which 29 metabolites were unique to AHI metabolite index. Coefficients for all metabolites from LASSO trained in gender-combined and gender-stratified samples are provided in the Supplementary Information (Supplementary Table S18). The summation of selected metabolites and their beta coefficients from LASSO were then z-scored to generate the metabolite index (study mean and SD used in z-score is provided in Supplementary Table S19).

### Metabolite indices associations with OSA and AHI in HCHS/SOL and in independent validation in MESA

**Figure 4:**
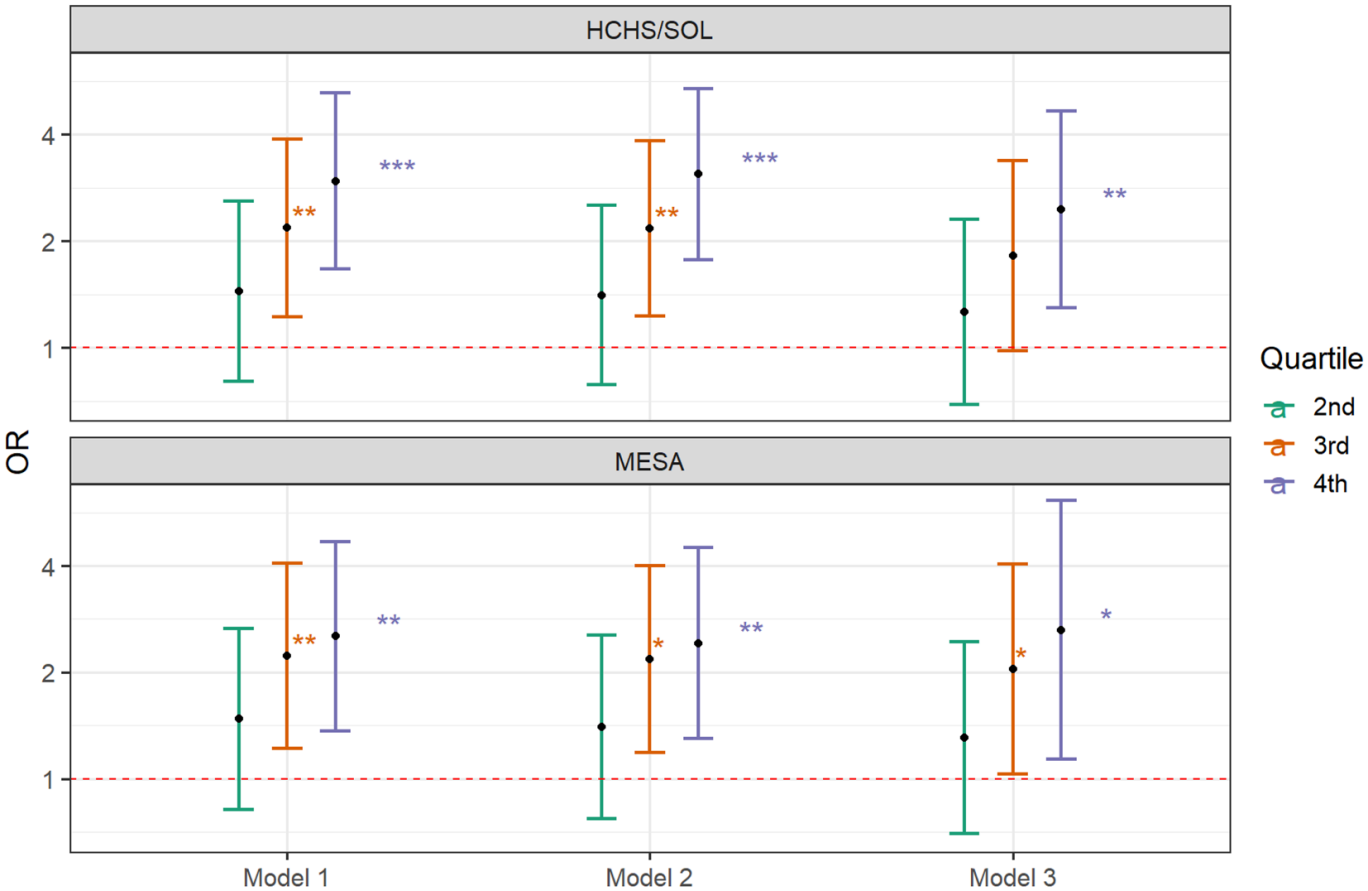
Odds ratio of metabolite index for OSA by quartiles in HCHS/SOL and MESA. *indicates p < 0.05. **indicates p < 0.01. ***indicates p < 0.001 In HCHS/SOL: Model 1 adjusted for age, gender, center, background, and BMI. Model 2 adjusted for age, gender, center, background, BMI, alcohol use, smoking status, physical activity and diet (AHEI 2010). Model 3 adjusted for age, gender, center, background, BMI, alcohol use, smoking status, physical activity, diet, T2DM, hypertension, fasting glucose, fasting insulin, HOMA IR, HDL, LDL, total cholesterol, triglycerides, systolic blood pressure and diastolic blood pressure. In MESA: Model 1 adjusted for age, gender, BMI, study site (site WFU and UCLA are combined due to low cell count), and race. Model 2 adjusted for age, gender, BMI, study site, race, alcohol use and smoking status. Model 3 adjusted for age, gender, BMI, study site, race, alcohol use, smoking status, hypertension indicator, fasting glucose, HDL, LDL, cholesterol, triglycerides, systolic blood pressure and diastolic blood pressure.

We constructed OSA and AHI metabolite indices in both HCHS/SOL and MESA based on the weights from the LASSO regressions conducted in HCHS/SOL. Table 3 provides overall and gender-stratified results. As expected by construction, the metabolite indices were associated with their phenotypes in HCHS/SOL (OSA metabolite index OR: 1.50; 95% CI 1.30–1.74; *p* < 0.001; AHI metabolite index beta = 2.12 per 1 SD of the index, 95% CI 1.43–2.81; *p* < 0.001) after adjustment for demographic covariates (i.e. age, gender, BMI, study center, and Hispanic/Latino background); the associations persisted after additional adjustment of lifestyle (i.e. alcohol usage, smoking status, physical activity and diet) and comorbidity (i.e. diabetes, hypertension, fasting glucose, fasting insulin, HOMA-IR, HDL, LDL, total cholesterol, triglycerides, systolic blood pressure, and diastolic blood pressure) covariates (OSA metabolite index OR: 1.50; 95% CI 1.21–1.85; *p* < 0.001; AHI metabolite index beta = 1.73 per 1 SD of the index, 95% CI 0.96–2.50; *p* < 0.001). In the validation dataset, the OSA metabolite index was also associated with an increased odds ratio for OSA (OR: 1.41; 95% CI 1.13–1.77; *p* = 0.003) (Table 3) when adjusted for demographic (i.e. age, gender, BMI, study site, and race) covariates and remained similar when adjusted for lifestyle (i.e. alcohol usage and smoking status) and comorbidity (i.e. hypertension, fasting glucose, HDL, LDL, cholesterol, triglycerides, systolic blood pressure and diastolic blood pressure) covariates (OR: 1.55; 95% CI 1.10–2.20; *p* = 0.013). When compared with the lowest quartile of the OSA metabolite index, the top quartile showed more than a two-fold increase in odds for OSA [OR: 2.53; 95% CI (1.37–4.70); *p* = 0.003] in the primary model and remained significant when adjusted for lifestyle and comorbidity covariates [OR: 2.63; 95% CI (1.14–6.14); *p* = 0.024] (see Fig. 4 and Supplementary Table S20). AHI metabolite index associations had higher p-values in MESA, compared to OSA metabolite index associations (Table 3). The AHI metabolite index was only replicated in women, adjusted for demographic, lifestyle, and comorbidity covariates in MESA. Notably, both OSA metabolite index and AHI metabolite index associations with their respective phenotypes were stronger when evaluated in women compared to the overall sample, in both HCHS/SOL and MESA.

**Table 3:** Estimated associations between OSA and AHI metabolite indices and their respective phenotypes, in HCHS/SOL and MESA.

OSA	HCHS/SOL						MESA					
	Both		Male		Female		Both		Male		Female	
*Predictors*	OR*	*p*	*OR*	*p*	*OR*	*p*	*OR*	*p*	*OR*	*p*	*OR*	*p*
**Model 1**†												
OSA-Met index	1.50 [1.30–1.74]	** < 0.001**	1.41 [1.17–1.70]	** < 0.001**	1.78 [1.47–2.14]	** < 0.001**	1.41 [1.13–1.77]	**0.003**	1.17 [0.81–1.72]	0.403	1.48 [1.11–2.00]	**0.009**
Male-specific OSA-Met index			1.53 [1.27–1.83]	** < 0.001**					1.24 [0.87–1.77]	0.234		
Female-specific OSA-Met index					4.37 [2.73–7.01]	** < 0.001**					1.40 [1.04–1.90]	**0.029**
**Model 2**‡												
OSA-Met index	1.53 [1.33–1.77]	** < 0.001**	1.44 [1.20–1.72]	** < 0.001**	1.86 [1.52–2.27]	** < 0.001**	1.39 [1.11–1.76]	**0.004**	1.15 [0.78–1.69]	0.476	1.46 [1.09–1.98]	**0.013**
Male-specific OSA-Met index			1.55 [1.29–1.87]	** < 0.001**					1.23 [0.86–1.78]	0.249		
Female-specific OSA-Met index					5.10 [3.15–8.27]	** < 0.001**					1.42 [1.05–1.94]	**0.024**
**Model 3**§												
OSA-Met index	1.50 [1.21–1.85]	** < 0.001**	1.43 [1.09–1.88]	**0.011**	1.89 [1.35–2.64]	** < 0.001**	1.55 [1.10–2.20]	**0.013**	1.30 [0.74–2.30]	0.365	1.71 [1.07–2.78]	**0.026**
Male-specific OSA-Met index			1.56 [1.25–1.95]	** < 0.001**					1.18 [0.77–1.82]	0.455		
Female-specific OSA-Met index					7.25 [3.89–13.52]	** < 0.001**					1.43 [0.98–2.11]	0.069

AHI	HCHS/SOL						MESA					
	Both		Male		Female		Both		Male		Female	
*Predictors*	Estimates	*p*	Estimates	*p*	Estimates	*p*	Estimates	*p*	Estimates	*p*	Estimates	*p*
**Model 1**†												
AHI-Met Index	2.12 [1.43–2.81]	** < 0.001**	2.39 [1.21–3.58]	** < 0.001**	1.47 [1.00–1.94]	** < 0.001**	1.45 [− 0.19–3.08]	0.083	− 1.59 [− 4.33–1.15]	0.254	3.38 [1.42–5.33]	**0.001**
Male-specific AHI-Met Index			3.22 [2.11–4.34]	** < 0.001**					− 4.99 [-24.53–14.54]	0.615		
Female-specific AHI-Met Index					1.69 [1.20–2.18]	** < 0.001**					2.08 [0.10–4.06]	**0.040**
**Model 2**‡												
AHI-Met Index	2.12 [1.41–2.83]	** < 0.001**	2.41 [1.22–3.61]	** < 0.001**	1.49 [1.01–1.96]	** < 0.001**	1.42 [− 0.22–3.06]	0.089	− 1.55 [− 4.32–1.21]	0.269	3.25 [1.30–5.21]	**0.001**
Male-specific AHI-Met Index			3.23 [2.11–4.35]	** < 0.001**					− 3.48 [− 23.25–16.3]	0.729		
Female-specific AHI-Met Index					1.73 [1.24–2.23]	** < 0.001**					1.92 [− 0.07–3.91]	0.058
**Model 3**§												
AHI-Met Index	1.73 [0.96–2.50]	** < 0.001**	2.14 [0.79–3.48]	**0.002**	1.11 [0.64–1.58]	** < 0.001**	1.20 [− 0.56–2.96]	0.181	− 0.96 [− 3.96–2.03]	0.527	2.65 [0.54–4.76]	**0.014**
Male-specific AHI-Met Index			3.09 [1.93–4.26]	** < 0.001**					− 0.78 [− 21.45–19.9]	0.941		
Female-specific AHI-Met Index					1.35 [0.91–1.79]	** < 0.001**					1.55 [− 0.59–3.69]	0.154

*per 1 STD increase in metabolite index (Met Index).

†Model 1 adjusted for age, gender, BMI, study center, and Hispanic/Latino background in HCHS/SOL, and adjusted for age, gender, BMI, study site (site WFU and UCLA are combined due to low cell count), and race in MESA.

‡Model 2 adjusted for all covariates in model 1, in HCHS/SOL model 2 additionally adjusted for alcohol usage, smoking status, physical activity and diet; in MESA, model 2 additionally adjusted for alcohol usage and smoking status.

§Model 3 adjusted for all covariates in model 2, in HCHS/SOL, model 3 additionally adjusted for diabetes, hypertension, fasting glucose, fasting insulin, HOMA-IR, HDL, LDL, total cholesterol, triglycerides, systolic blood pressure, diastolic blood pressure; in MESA, model 3 additionally adjusted for hypertension indicator, fasting glucose, HDL, LDL, cholesterol, triglycerides, systolic blood pressure and diastolic blood pressure.

Significant values are in bold.

Results from the secondary analyses of gender-specific metabolite indices are also provided in Table 3. Only the female-specific metabolite indices were replicated in MESA in models 1 and 2, but their associations with sleep apnea traits in women were weaker than that of the metabolite indices trained on the full HCHS/SOL sample, both in terms of *p*-value and of estimated effect size.

When adjusted for the time interval between the sleep exam and the blood collection in MESA, the estimated effect sizes and *p*-values of the associations between OSA and metabolite index did not substantially change, in any model (Supplementary Table S21). When limiting the MESA study sample to only Hispanics, the associations were slightly attenuated except in model 3 (Supplementary Fig. S2). The associations between the OSA LASSO metabolite index and other sleep disordered breathing phenotypes showed consistent directionality with its OSA association (Supplementary Fig. S3) – positively associated with typical OSA severity measures (e.g. pctlt90) while inversely associated with minimum and average oxyhemoglobin saturation. There was no evidence of association of the metabolite index with average respiratory event length. In MESA, the OSA metabolite index was associated with average and minimum oxygen saturation but its associations with other phenotypes were weaker.

Two additional metabolite indices were constructed for OSA based on the 7 significant SMA discovery results. The SMA metabolite index constructed with coefficients extracted directly from the SMA analysis as weights did not show as strong associations with OSA in HCHS/SOL or MESA, while the SMA-GLM metabolite index with coefficients extracted from the joint unpenalized generalized linear regression using the 7 metabolites together showed comparable effect size estimates to the LASSO-based OSA metabolite index (Supplementary Table S21, Supplementary Fig. S2). Parameters for constructing the SMA and the SMA-GLM metabolite indices (i.e. metabolite coefficients, mean and SD for z-scoring) are provided in Supplementary Table S22; the correlation matrix of the 7 metabolites is provided in Supplementary Fig. S4.

## Discussion

In this paper, we leveraged metabolomics data from two large, diverse community-based cohorts to derive the first metabolite index for moderate to severe OSA, as well as to identify individual metabolites associated with this disorder. We studied 219 metabolites and their associations with OSA and AHI in the HCHS/SOL using two methods: (1) analysis of individual metabolites, and (2) LASSO to identify a subset of metabolites that jointly predicted OSA or AHI. Then, we studied the associations in an independent validation study, MESA. We used the results from LASSO to derive OSA and AHI metabolite indices. In MESA, the OSA metabolite index was significantly associated with its respective phenotype, moderate to severe OSA; e.g., individuals in the highest quartile for OSA metabolite index had a more than twofold increased odds of moderate to severe OSA- both in the derivation sample and in an independent sample that varied by ancestry, age, and OSA prevalence. Findings persisted after adjusting for multiple lifestyle and health covariates. In contrast, when modeling AHI as a continuous measure of sleep apnea, weaker associations were observed, except for the top quartiles among females. In the association analysis of individual metabolites, seven metabolites were associated with OSA in HCHS/SOL (FDR *p* < 0.05), of which four associations were replicated in MESA.

We implemented two main approaches to study the metabolomic correlates of sleep apnea phenotypes: LASSO and individual-metabolite regression analysis. These approaches serve different purposes: single metabolite regression highlights individual metabolites associated with sleep apnea phenotypes without adjustment to other metabolites, while LASSO estimates the combined effect of multiple metabolites. A metabolite identified as associated with sleep apnea phenotypes individually may not be selected by the LASSO analysis (e.g. if a different metabolite correlated to it was selected by LASSO). Thus, it is not surprising that two of the replicated metabolites identified in single metabolite analysis were not selected by LASSO. Similarly, a specific metabolite may be selected by LASSO, but not by individual metabolite analysis due to adjustment for multiple testing, which is not done in the LASSO analysis. The OSA metabolite index, constructed based on the LASSO results, is a single index using multiple blood biomarkers which together reflect the biochemical differences in the blood of individuals with and without OSA. The OSA metabolite index also showed stronger association with OSA than any single metabolite, in both the discovery and validation study, consistent with the influence of multiple metabolites in OSA pathophysiology (see Table 3, Supplementary Table S3). When comparing the three metabolite indices, the SMA metabolite index did not perform as well, likely due to correlations among the metabolites (Supplementary Fig. S4), while the SMA-GLM metabolite index is comparable to the LASSO-based metabolite index (Supplementary Table S3). While future work is needed to study whether the metabolite index can be used in the clinic for screening or clinical management of patients with OSA, the study results supports its statistical and epidemiological utility given that the metabolite index had higher statistical power than analyses testing single metabolite associations. These indices, providing biologically relevant and novel associations with OSA, may enable additional studies of the pathophysiology of OSA and its relationship with other cardiometabolic conditions. However, additional research is needed to understand the best analytical strategy (i.e., one step approach like LASSO or two-step approach like SMA-GLM).

Four metabolites were replicated in the single metabolite regression: glutamate, oleoyl-linoleoyl-glycerol (18:1/18:2) (DAG(36:3)), linoleoyl-linoleoyl-glycerol (18:2/18:2) (DAG(36:4)) and phenylalanine, among which glutamate and phenylalanine remained positively associated with OSA after adjusting for lifestyle and comorbidities in addition to basic demographics. Both metabolites have some previous evidence linking them to OSA or other sleep disorders, as well as cardiometabolic diseases, and suggest that elevations in glutamate and phenylalanine can be investigated as biomarkers for adverse outcomes in patients with OSA. High plasma glutamate has been associated with OSA-related health factors, including total and visceral adiposity, dyslipidemia and insulin resistance^27^, as well as increased risks for incident cardiovascular disease^28^, type 2 diabetes^29^, and subclinical atherosclerosis^30^, independent of established cardiovascular risk factors. The positive association between glutamate and OSA observed in our study may suggest a shared metabolomic profile for OSA and other cardiometabolic phenotypes. The sources for elevated glutamate are unclear but previous studies in rats animals and human showed that more frequent apneas during sleep led to an increased level of glutamate in brain^31–33^. Glutamate is the major excitatory neurotransmitter in the brain, and modulates brain energy metabolism and neuronal synaptic plasticity. Although the blood–brain barrier prevents plasma glutamate from freely permeating into the central nervous system^34^, when glutamate increases in the brain, the brain-to-blood glutamate efflux also increases, as suggested by the correlation between the peripheral glutamate and the central nerve system glutamate levels^35^. These findings support further research addressing the roles of peripheral and central glutamate in the pathophysiology of OSA.

Phenylalanine is an essential aromatic amino acid that plays a key role in the biosynthesis of other amino acids, including the neurotransmitters, dopamine and norepinephrine. Studies have shown that plasma phenylalanine level can be elevated due to inflammation^36,37^, which is a common finding in OSA^38^. One mechanism for the increased levels of phenylalanine may be through chronic hypoxia, which has been reported to increase both systemic and cerebral delivery of phenylalanine^39^. This is in line with our evidence: peripheral phenylalanine was elevated among individuals with moderate to severe OSA (Supplementary Table S3). A prior lab-based study that measured a few metabolites over the course of sleep reported that phenylalanine levels decreased less overnight among patients with OSA compared to controls^40^. Phenylalanine levels were also reported to be elevated after sleep restriction^41^. The downstream effects of phenylalanine have been studied more widely in other chronic conditions, with reports of associations with elevated pro-inflammatory cytokines, suppressed immunity and increased mortality among heart failure patients^42^, and with more rapid telomere shortening consistent with accelerated aging^43^. Recent studies reported elevations of phenylalanine associated with chronic obstructive lung disease severity^44^, which was postulated to reflect muscle breakdown and respiratory muscle insufficiency^45^. Elevated plasma phenylalanine was shown to be a strong predictor for cardiovascular risk^46^, and a biomarker, mediator and potentially therapeutic target for pulmonary hypertension^47^. Further research on the association of OSA and phenylalanine may further identify the roles of hypoxia, inflammation, and muscle function in the pathophysiology of OSA and cardiometabolic conditions.

Our study also demonstrated increased plasma levels of two diacylglycerols (DAGs): DAG(36:3) and DAG(36:4) occurred in association with moderate to severe OSA. Altered lipids metabolism is often observed among patients with OSA^48–50^; specifically, intermittent hypoxemia can stimulate lipolysis, increasing free fatty acid levels^51^. Abnormalities in lipid metabolism may result in liver and skeletal muscle fat deposition, exacerbating OSA through inflammatory or muscle-related pathways^52^. Therefore, the associations with these diacylglycerols may reflect mechanisms by which OSA related hypoxemia alters fatty acid metabolism. These associations, however, did not replicate in the validation study once adjusted for comorbidities, suggesting that the associations might be confounded by cardiometabolic conditions that often accompany OSA.

Estimated OSA associations, in both LASSO and single metabolite analysis, were generally stronger than AHI associations. Potential reasons are the variability of AHI across its continuum (with high night to night variability noted at low to mildly elevated levels) and the potential non-linear metabolomic associations with AHI not easily modeled in these analyses. Notably, non-linearity (i.e. a threshold effect) was previously shown for AHI association with hypoxemia and sympathetic nervous system activation burden^53^.

Gender differences have been increasingly reported among individuals with OSA^17^. Population-based studies have shown that prevalence of OSA is higher among men than women, particularly in pre-menopausal women^54^. However, some data indicate that metabolic syndrome and cardiovascular conditions are more strongly associated with OSA among female patients compared to males^55,56^. Indeed, when assessing the metabolite indices developed in both genders and tested for their associations with OSA and AHI, we observed stronger associations among women than men in the MESA validation dataset (Table 3). However, compared to the metabolite indices developed in combined gender strata, gender-specific metabolite indices had weaker associations with the OSA/AHI in the validation data set (weaker effect size estimates and higher p-value), which may be the result of lower statistical power when using a smaller dataset for discovery or differences in the age distributions of the two samples.

In addition to pointing to novel individual metabolites that play a role in OSA, our metabolite indices also showed moderately strong associations with OSA in an external sample, despite the marked differences in race/ethnicity, age, and OSA prevalence compared to the discovery sample. We found no evidence to support the superiority of the index in a sample restricted to Hispanic individuals (Supplementary Fig. S2 and Supplementary Table S21). This supports the overall generalizability of the metabolite index across diverse populations. Nonetheless, the utility of a twofold increased risk of OSA among individuals in the highest metabolite index quartile in helping to screen or triage patients for more comprehensive testing will need to be formally evaluated, potentially combining metabolite data with other information, such as OSA-related symptoms, to improve screening.

A strength of our study is that our population-based sample is more than tenfold larger than prior studies^57^, includes a high proportion of ethnic/racial minorities who have been under-represented in research but are at increased for adverse health outcomes, and is more representative of samples in the general population who remain include large numbers of undiagnosed individuals. We used rigorous statistical methods, adjusted for a large number of lifestyle and health covariates, and were able to replicate the main findings despite large differences in our discovery and validation populations, which suggests relatively strong associations and generalizability of the metabolite associations with OSA.

There are several limitations in this study. The temporal relationship between the blood sample collection and sleep test was concurrent in HCHS/SOL and on average one year apart in MESA, allowing for cross-sectional associations, but limiting our ability to discern causal pathways. The associations between OSA and the metabolite index did not substantively change even after controlling for the time interval between blood draw and sleep test in MESA, suggesting the metabolite profiling for OSA is stable (Supplementary Tables S19, S3). Although over 1000 metabolites were quantified in both populations, less than 300 metabolites were matched between the two platforms (after quality control only 219 distinct metabolites were mapped). We limited our study to only the matched metabolites to allow for replication testing, which strengthens the results and conclusions (results on full set of metabolites are provided in Supplementary Tables S12–S17). MESA metabolomic profiling was conducted using three complementary platforms measuring several broad classes of small molecules therefore multiple chemical compounds from MESA were mapped to the same metabolite in the HCHS/SOL. We chose a single feature to map to any HCHS/SOL metabolite based on a set of rules related presence of redundant ions, data missingness and skewness. In the future, other more optimal approaches may be proposed and studied. Some associations failed to replicate in MESA, potentially due to heterogeneity in different populations and low power in MESA, which had a small sample size. Finally, the definitions of AHI differed slightly in the two studies: while the 3% oxyhemoglobin desaturation criterion applied to hypopneas only in MESA, due to differences in the recording montage, a 3% desaturation criterion was applied for all respiratory events in HCHS/SOL.

It is important to consider how to use, interpret, and transfer metabolite indices that combine multiple metabolites across studies. First, different from typical biomarkers, values of a metabolite index (as constructed in this work) does not have any absolute reference, due to the standardization of the individual metabolite concentrations and of the final weighted index. Thus, metabolite index of 0 implies the population average. Further, metabolites are summed with potentially both negative and positive weights, accounting for the fact that some are positively, and some are negatively associated with increased OSA risks. The final weighted sum is always associated with increased OSA risks. Second, it is worth noting that the metabolite indices sum metabolites that were rank-normalized within studies. If targeted metabolomics analyses were used, it may have been more appropriate to not rank-normalize or to apply standardization in a different way. More work is needed to learn how to transfer metabolite indices constructed based on untargeted metabolomics survey to targeted measurements. Finally, we reported metabolite indices associations in HCHS/SOL and in MESA while standardizing the indices in each study separately. As the metabolite indices are sums of normalized metabolites in the sample, their mean is close to zero, but may be slighted different than zero based on the specific sample used. Their variance depends not only on the variance of each (normalized) metabolite, but also on the correlation between metabolites in the sample, which may cause differences in scale of metabolite indices before z-scoring between studies. While z-scoring metabolite indices does not affect the strength of associations, it does affect the estimated effect sizes. Reporting of associations and risk by in-sample quartiles, in contrast, does not depend on scales and z-scoring, but also suffers from limited transferability, as one person who may be in a low quartile in one set of samples may be in a higher quartile in a different set of samples. More work is needed to develop a framework for transferability of metabolite indices across studies.

In summary, we used two large datasets of population-based multi-ethnic cohort studies to study metabolomics associations with OSA. We developed metabolite indices that replicated across datasets, and had a statistically significant association with OSA even after adjusting for multiple lifestyle factors and cardiometabolic comorbidities. In future work we will study the possibility of developing an OSA screening tool based on metabolite indices. Four metabolites also replicated in an independent dataset, of which one was previously implicated in OSA, and two were previously connected to sleep disorders. Collectively, our findings support the utility of metabolomic profiling for generating metabolite indices of sleep apnea in racially and ethnically diverse populations, and its potential to provide insight into the pathophysiology of OSA.

## Supplementary Information

Supplementary Information 1.

Supplementary Tables.

Supplementary Figures.

Supplementary Legends.

## Data Availability

In accordance with participants informed consent, MESA and HCHS/SOL data are available through data use agreement in dbGaP according to the study specific accessions. MESA phenotypes are available in: phs000209; MESA metabolomics data have been deposited and will become available in: phs001416; and HCHS/SOL phenotypes: phs000810. HCHS/SOL metabolomics data are available via data use agreement with the HCHS/SOL Data Coordinating Center at the University of North Carolina at Chapel Hill, see collaborators website: https://sites.cscc.unc.edu/hchs/. All data generated or analyzed during this study are included in this published article and its supplementary information file. In addition, summary statistics from single metabolite association analyses in MESA and HCHS/SOL for all studied metabolites are provided as Supplementary Information.
