# Supplementary material for: Development and validation of a metabolite index for obstructive sleep apnea across race/ethnicities
Source: Sci Rep. 2022 Dec 16;12:21805. doi: 10.1038/s41598-022-26321-9 (PMC9758170; doi:10.1038/s41598-022-26321-9)

Supplementary Figures

Contents

[Supplementary Figure S1. Flowchart of the study population selection and metabolomic data preprocessing 2](#_Toc120252796)

[Supplementary Figure S2. Estimated odds ratios of metabolite indices for OSA in HCHS/SOL and MESA 3](#_Toc120252797)

[Supplementary Figure S3. Associations between OSA LASSO metabolite index and other sleep disordered breathing phenotypes in HCHS/SOL and MESA 4](#_Toc120252798)

[Supplementary Figure S4. Correlation matrix of the metabolites with FDR p<0.05 in the single metabolite association analysis with OSA in HCHS/SOL 5](#_Toc120252799)

# Supplementary Figure S1. Flowchart of the study population selection and metabolomic data preprocessing

HCHS/SOL
Obs: n=3978; Metabolites: n=1138

Obs: n=3968; Metabolites: n=1043
(899 continuous + 142 dichotomized)

Excluded samples >=25% missing
metabolites >=75% missing

Excluded unknown metabolites

Obs: n=3968; Metabolites: n=706
(639 continuous + 67 dichotomized)

Obs: n=3968; Metabolites: n=219
(209 continuous + 10 dichotomized)

Excluded unmatched metabolites between HCHS and MESA

Excluded samples with missing sleep traits

Obs: n=3507; Metabolites: n=219
(209 continuous + 10 dichotomized)

# Supplementary Figure S2. Estimated odds ratios of metabolite indices for OSA in HCHS/SOL and MESA


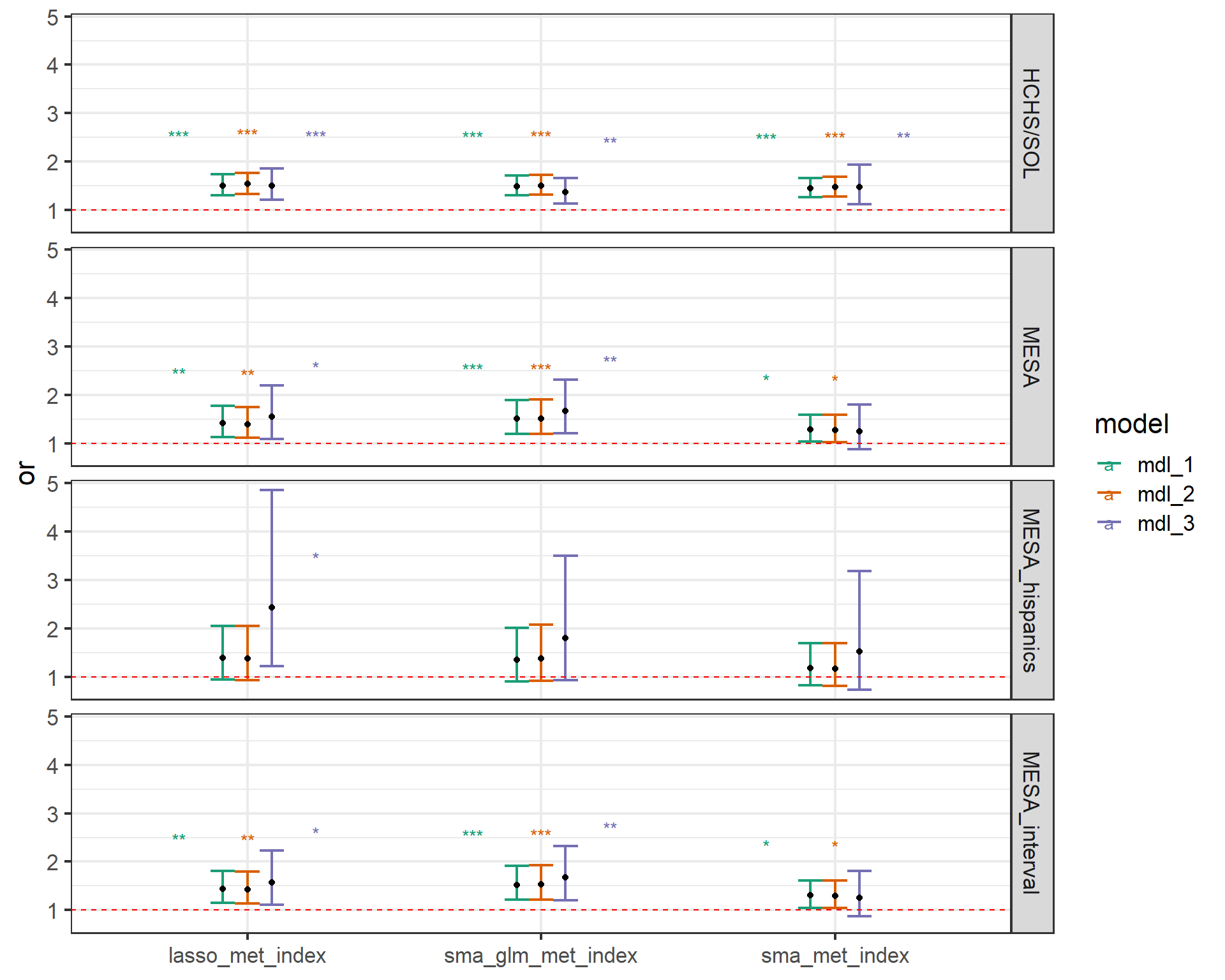


* indicates *p*<0.05. ** indicates *p*<0.01. *** indicates *p*<0.001

In HCHS/SOL: Model 1 adjusted for age, gender, center, background, and BMI. Model 2 adjusted for age, gender, center, background, BMI, alcohol use, smoking status, physical activity and diet (AHEI 2010). Model 3 adjusted for age, gender, center, background, BMI, alcohol use, smoking status, physical activity, diet, T2DM, hypertension, fasting glucose, fasting insulin, HOMA IR, HDL, LDL, total cholesterol, triglycerides, systolic blood pressure and diastolic blood pressure. In MESA: Model 1 adjusted for age, gender, BMI, study site (site WFU and UCLA are combined due to low cell count), and race. Model 2 adjusted for age, gender, BMI, study site, race, alcohol use and smoking status. Model 3 adjusted for age, gender, BMI, study site, race, alcohol use, smoking status, hypertension indicator, fasting glucose, HDL, LDL, cholesterol, triglycerides, systolic blood pressure and diastolic blood pressure.

MESA_hispanics: only Hispanics were included in the analysis; MESA_interval: the time differences between the sleep exam and the blood collection were adjusted in all models.

lasso_met_index: LASSO-based metabolite index; sma_glm_met_index: Generalized linear regression model based- and single metabolite association analysis guided metabolite index; sma_met_index: single metabolite association analysis based metabolite index

# Supplementary Figure S3. Associations between OSA LASSO metabolite index and other sleep disordered breathing phenotypes in HCHS/SOL and MESA


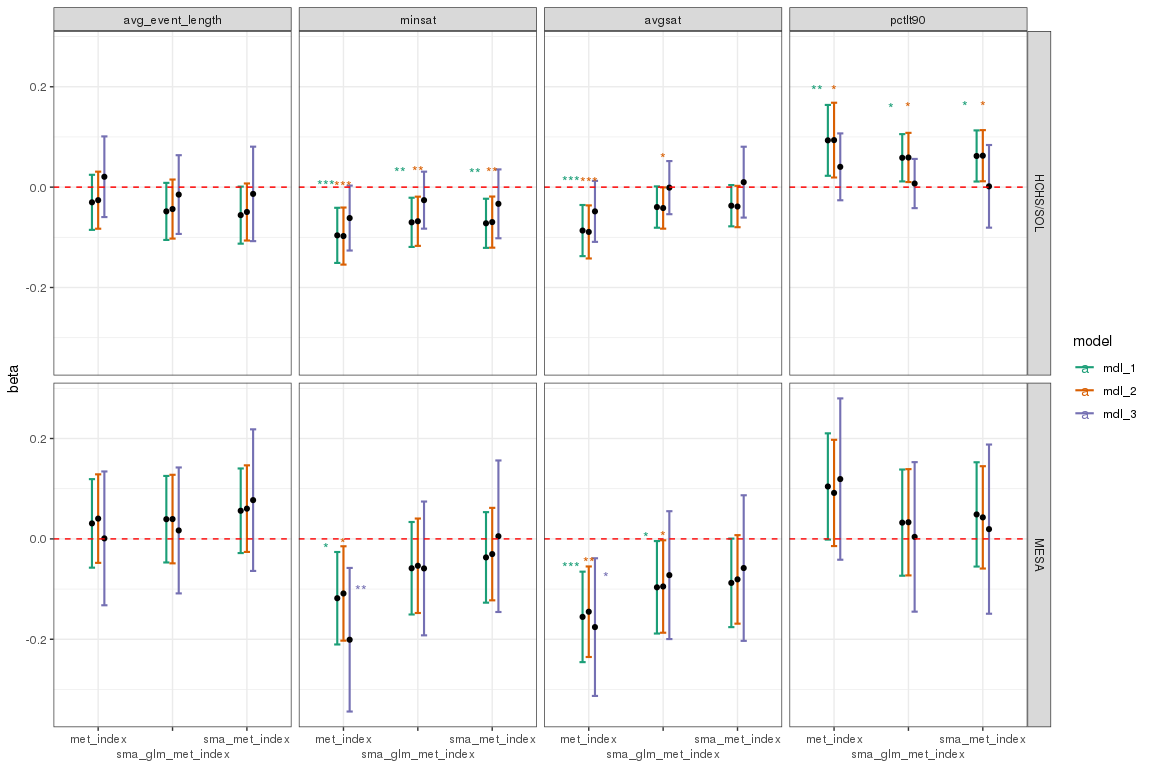


* indicates *p*<0.05. ** indicates *p*<0.01. *** indicates *p*<0.001

In HCHS/SOL: Model 1 adjusted for age, gender, center, background, and BMI. Model 2 adjusted for age, gender, center, background, BMI, alcohol use, smoking status, physical activity and diet (AHEI 2010). Model 3 adjusted for age, gender, center, background, BMI, alcohol use, smoking status, physical activity, diet, T2DM, hypertension, fasting glucose, fasting insulin, HOMA IR, HDL, LDL, total cholesterol, triglycerides, systolic blood pressure and diastolic blood pressure. In MESA: Model 1 adjusted for age, gender, BMI, study site (site WFU and UCLA are combined due to low cell count), and race. Model 2 adjusted for age, gender, BMI, study site, race, alcohol use and smoking status. Model 3 adjusted for age, gender, BMI, study site, race, alcohol use, smoking status, hypertension indicator, fasting glucose, HDL, LDL, cholesterol, triglycerides, systolic blood pressure and diastolic blood pressure.

avg_event_length: average respiratory event length; avgsat: average oxygen saturation; hb: hypoxic burden; minsat: minimum oxygen saturation; pctlt90: the percentage of sleep time with oxyhemoglobin saturation below 90%.

# Supplementary Figure S4. Correlation matrix of the metabolites with FDR p<0.05 in the single metabolite association analysis with OSA in HCHS/SOL


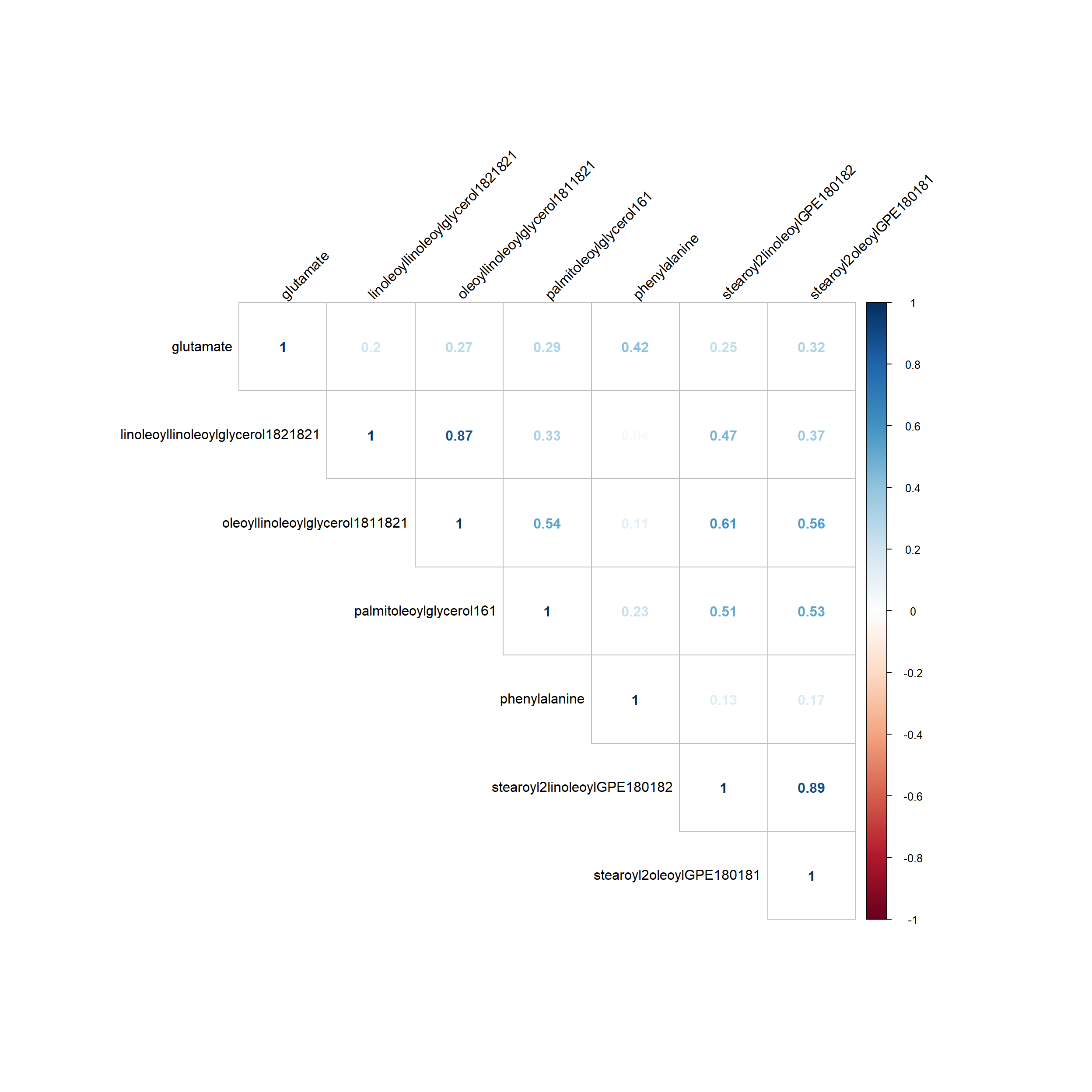

Supplement: Supplementary file 3 — Supplementary Figures. [file 41598_2022_26321_MOESM3_ESM.docx]
