# Supplementary material for: Development and validation of a metabolite index for obstructive sleep apnea across race/ethnicities
Source: Sci Rep. 2022 Dec 16;12:21805. doi: 10.1038/s41598-022-26321-9 (PMC9758170; doi:10.1038/s41598-022-26321-9)
Supplement: Supplementary file 4 — Supplementary Legends. [file 41598_2022_26321_MOESM4_ESM.docx]

Supplementary Information

Supplementary File 1. R code for constructing metabolite indices

Supplementary File 2. Supplementary Tables S1-S22

Supplementary File 3. Supplementary Figures S1-S4.
